# Turning the tides: achieving rapid and safe glucose control in adolescents with suboptimally controlled type 1 diabetes using advanced hybrid closed loop systems

**DOI:** 10.3389/fendo.2024.1243565

**Published:** 2024-04-02

**Authors:** Valeria Castorani, Andrea Rigamonti, Giulio Frontino, Elisa Morotti, Federica Sandullo, Francesco Scialabba, Francesca Arrigoni, Benedetta Dionisi, Riccardo Foglino, Camilla Morosini, Gabriele Olivieri, Riccardo Bonfanti

**Affiliations:** ^1^ Department of Pediatrics, Pediatric Diabetes Unit, IRCCS San Raffaele Scientific Institute, Milano, Italy; ^2^ Diabetes Research Institute, IRCCS San Raffaele Scientific Institute, Milano, Italy

**Keywords:** Type 1 diabetes (or diabetes), HbA1c (A1C), glucose risk index, adolescence, time in range (TIR), Automated insulin delivery (AID)

## Abstract

**Aim:**

Many adolescents with T1D experience a decline in metabolic control due to erratic eating habits and subpar adherence to treatment regimens. The objective of our retrospective observational study was to assess the effect of the Tandem Control IQ (CIQ) advanced hybrid closed-loop (AHCL) system on a cohort of adolescents with suboptimal glucose control.

**Methods:**

We retrospectively evaluated 20 non-adherent patients with T1D, who were inconsistently using Multiple Daily Injections (MDIs) and flash glucose monitoring and were subsequently started and on CIQ. Glucometrics and the Glucose Risk Index were assessed at baseline and after 2 weeks, 1 month, and 6 months of CIQ use.

**Results:**

The study included 20 adolescents with T1D (HbA1c: 10.0% ± 1.7). Time in range (TIR) increased from 27.1% ± 13.7 at baseline to 68.6% ± 14.2 at 2 weeks, 66.6% ± 10.7 at 1 month, and 60.4% ± 13.3 at 6 months of CIQ use. Time above range (TAR) >250 mg/dL decreased from 46.1% ± 23.8 to 9.9% ± 9.5 at 2 weeks, 10.8% ± 6.1 at 1 month, and 15.5% ± 10.5 at 6 months of AHCL use. Mean glucose levels improved from 251 mg/dL ± 68.9 to 175mg/dL ± 25.5 after 6 months of CIQ use. The Glucose Risk Index (GRI) also significantly reduced from 102 to 48 at 6 months of CIQ. HbA1c also improved from 10.0% ± 1.7 at baseline to 7.0% ± 0.7 after 6 months. Two patients experienced a single episode of mild diabetic ketoacidosis (DKA).

**Conclusions:**

AHCL systems provide a significant, rapid, and safe improvement in glucose control. This marks a pivotal advancement in technology that primarily benefited those who were already compliant.

## Research in context

### Evidence before this study

Advanced hybrid closed-loop (AHCL) systems are known to improve glycemic control in individuals with type 1 diabetes (T1D). However, the efficacy of these systems has not been extensively studied in the specific population of non-compliant adolescents who struggle with suboptimal glucose control and were previously using multiple daily injections.

### Added value of this study

Our study examined the impact of the Tandem Control IQ (CIQ) AHCL system in a cohort of 20 non-compliant adolescents with T1D over 6 months. We found that there was a swift and substantial improvement in time in range (TIR), decrease in time above range (TAR), and a reduction in mean glucose levels with the use of the AHCL system. Interestingly, these positive changes were seen as early as 2 weeks into use of the CIQ system, demonstrating a rapid response to this form of treatment.

### Implications of all the available evidence

The results of this study suggest that AHCL systems can be highly beneficial for non-compliant adolescents with T1D, significantly improving their glucose profiles and reducing the risk of future complications. Despite the limitations of the study such as a small sample size and absence of a control group, our findings indicate that AHCL systems could be considered a first-line approach for this challenging group of patients. It is a testament to the potential of AHCL technology as a game changer, offering an improved quality of life and a future with fewer complications for this particular population. Further research with larger cohorts and longer follow-up periods will be useful to confirm and expand upon these findings.

## Introduction

Advanced hybrid closed-loop (AHCL) systems represent the next automation step, aiming to maximize normoglycemia by integrating continuous glucose monitoring with automated insulin delivery. Specifically, AHCL technology employs an algorithm that automatically modifies the basal insulin rate based on expected glucose levels, with automated bolus insulin correction for high glucose levels. Patients are only required to estimate carbohydrate consumption for meal boluses. These systems ensure that a significant percent of time is spent within the target glucose range, minimizing both hypo- and hyperglycemia events and significantly improving the quality of life for children with type 1 diabetes (T1D).

These systems represent the most recent available automatism in the treatment of T1D and, in a semi-automatic way, can independently regulate insulin delivery based on dynamic data from a glucose sensor; they are the Medtronic 780G system (Minimed Medtronic, Northridge, CA) and the Tandem Control IQ system (Tandem Inc., San Diego, CA).

Both Medtronic 780G and Tandem Control IQ, with their different algorithms, are equally effective in making possible a personalization of insulin therapy and an adaptation to the different needs of the subjects and their families (REF Schiaffini et al.).

While patients’ T1D management skills, such as carbohydrate counting, insulin dose calculations, and insulin-to-carbohydrate ratios, remain crucial components, the introduction of AHCL systems marks a shift towards optimal diabetes control and a significant reduction in patients’ self-management (1, 2).

Many adolescents with T1D may experience a deterioration in metabolic control due to erratic meal and exercise patterns, poor adherence to treatment regimens, hazardous and risk-taking behaviors, disordered eating behaviors, other mental health issues, and endocrine changes associated with puberty. These factors can lead to greater insulin resistance, resulting in suboptimal glycated hemoglobin (HbA1c) levels. As HbA1c levels during youth are highly predictive of long-term HbA1c trajectory, timely interventions are necessary to alter a life course predictive of premature development of diabetes complications (3).

Our retrospective observational study aims to evaluate the impact of the Tandem t:slim X2 Control IQ (CIQ) system (Tandem Diabetes Care, Inc.) in a cohort of diabetic adolescents with suboptimal glucose control.

## Methods

This retrospective, real-world, observational study using medical records included 20 patients with T1D and high-risk glycemia, using multiple day injections (MDIs) and flash glucose monitoring. All children met the American Diabetes Association (ADA) criteria for T1D diagnosis (4) with a current HbA1c of ≥8.5%. Exclusion criteria were medication indicating diabetes complications, systemic glucocorticoids, or any concomitant diseases that could interfere with glucometric parameters; patients with genetic disorders were also excluded. Appropriate informed consent/assent was obtained.

We included patients that were started on CIQ between June and December 2022. Carbohydrate counting was not included, as patients had previously expressed non-compliance.

Glucometrics, including time in range (TIR), time above range (TAR), time below range (TBR), glucose management indicator (GMI)%, mean sensor glucose with standard deviation (SD), coefficient of variation (CV), and Glycemia Risk Index (GRI), were evaluated at baseline and after 2 weeks, 1 month, and 6 months of CIQ use. HbA1c was also documented at baseline and after 6 months of CIQ technology.

Serious adverse events, including severe hypoglycemia and diabetic ketoacidosis (DKA), were registered during follow-up.

CGM and insulin data were collected from Tidepool platform. Statistical analyses was performed using SPSS version 23.0 software for Windows (SPSS Inc., Chicago, IL, USA). Values were expressed as mean ± standard deviations (SDs). A p-value <0.05 was considered statistically significant. Comparisons between groups were analyzed with independent samples *t*-test and Mann–Whitney test.

## Results

A total of 20 adolescents with T1D were included (mean age: 15.7 ± 1.9 years, 55% female). **Table 1** shows the baseline clinical and auxological characteristics of the study population.

**Table 1:** Clinical and auxological characteristics of study population at baseline.

Variables	Mean ± SD
**Sample size**	20
**Gender (Male/Female)**	9(45%)/11(55%)
**Age (years)**	15.7 ± 1.9
**Weight (kg) at baseline**	57.7 ± 12.7
**Height (cm) at baseline**	161.5 ± 10.0
**BMI (kg/m2) at baseline**	21.9 ± 3.3
**HbA1c (%)**	10.0 ± 1.7
**Disease duration at baseline (years)**	6.2 ± 4.0

**Figure 1:**
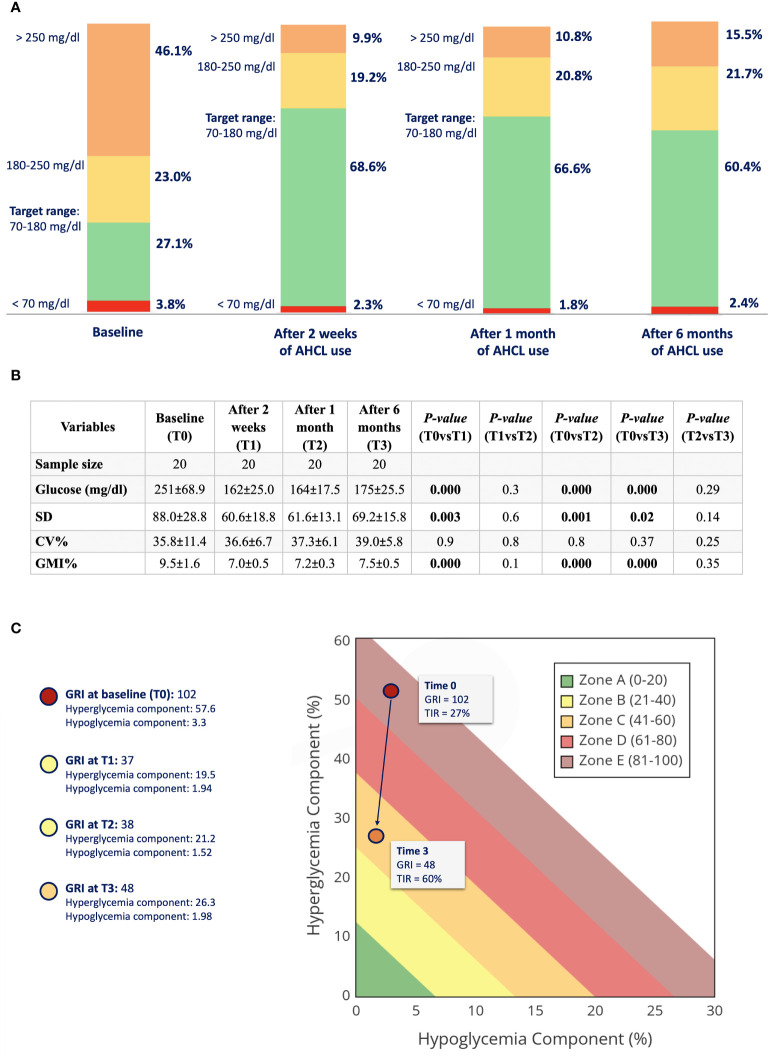
**(A)** Glucometric profile of the study population at baseline, after 2 weeks, 1 month, and 6 months of AHCL use. **(B)** Glucose oucomes of study population at baseline (TO), after 2 weeks (T1), after 1 month (2), and after 6 months (T3) of CIQ use. TIR, time in range; TAR, time above range; TBR, time below range; SD, standard deviation; CV, coefficient of variation; GMI, glucose management indicator. **(C)** GRI (Glycemia Risk Index) at baseline (TO) and after 6 months of CIQ technology.

During follow-up, TIR increased from 27.1% ± 13.7 at baseline to 68.6% ± 14.2 at 2 weeks (p<0.001), to 66.6% ± 10.7 at 1 month (p<0.001), and to 60.4% ± 13.3 at 6 months (p<0.001) of AHCL use. TAR >250 mg/dL decreased from 46.1% ± 23.8 to 9.9% ± 9.5 at 2 weeks (p<0.001), to 10.8% ± 6.1 at 1 month (p<0.001), and to 15.5% ± 10.5 at 6 months (p<0.001) using the CIQ system. No differences in TAR 180–250 mg/dL, TBR 54–70 mg/dL, or <54 mg/dL were found during follow-up (see **Figure 1A**). Mean glucose also improved from 251 mg/dL ± 68.9 to 162 mg/dL ± 25.0 after 2 weeks (p<0.001), to 164 mg/dL ± 17.5 after 1 month (p<0.001), and to 175 mg/dL ± 25.5 after 6 months (p<0.001) of follow-up. Accordingly, SD decreased from baseline (88.0 ± 28.8) to 2 weeks (60.6 ± 18.8) (p<0.005), 1 month (61.6 ± 13.1) (p=0.001), and 6 months (69.2 ± 15.8) (p=0.02) of follow-up. However, we did not find and statistically significant improvement in CV during follow-up. GMI% significantly reduced from baseline (9.5 ± 1.6%) (p<0.001) to 2 weeks (7.0 ± 0.5%) (p<0.001), 1 month (7.2 ± 1.6%) (p<0.001), and 6 months (7.5 ± 0.5%) (p<0.001) of CIQ use (see **Figure 1B**).

GRI, which closely corresponds to the clinician’s ranking of overall glycemia quality, reduced significantly from baseline to 6 months of CIQ technology (see **Figure 1C**). HbA1c also improve from 10 ± 1.7% at baseline to 7.0 ± 0.7% after 6 months of CIQ use (p<0.001).

No cases of severe hypoglycemia occurred during the study period. Two patients suffered from a single event of moderate DKA, likely due to infusion set occlusion. The events were resolved without complications.

## Discussion

Our study shows that non-compliant adolescents with T1D, previously using MDI therapy, may achieve a swift and sustained improvement in glucose profiles using AHCL systems. In particular, mean TIR improved by almost 40% within just 2 weeks of use, primarily accounted for by a significant reduction in time spent above 250 mg/dL. GRI drastically reduced, representing improved exposure to glucose excursions with CIQ technology. HbA1c, which remains one of the main predictors for chronic complications in people with diabetes, also significantly improved after 6 months.

During the 6-month follow-up, we documented only a slight but not statistically significant worsening of glucose control, likely due to patients’ poor adherence to treatment regimens over time, particularly with missed meal boluses.

The findings of the present case series align with previous studies using other advanced automated insulin delivery systems (5–8). This consistency of findings underscores the robustness of the AHCL algorithm and supports the application of closed-loop systems across a broad range of individuals with T1D. For the first time, the ADAPT study evaluated the clinical benefits of algorithm AHCL system in adults with T1D and suboptimal glucose control. In particular, the authors demonstrated that AHCL confers significant benefits in terms of glycemic control beyond those that can be achieved with multiple daily injections and suggest that AHCL should be considered at the early stages in the T1D treatment pathway (REF). Similarly, Lombardo et al. demonstrated the successful use of the AHCL system in a real-world study. The authors described the 6-month impact of the advanced automated functions of MiniMed™ 780G on GRI in a large cohort of children and adolescents with T1D also documenting the effectiveness and safety of AHCL technology in the pediatric population (REF).

Therefore, AHCL technology significantly, quickly, and safely improves glucose control, even in adolescents with poor glucose control, representing a turning point for technology that used to favor mainly those who were already compliant.

Our results, although possibly biased by the relatively short follow-up, suggest that even non-compliant adolescents with T1D can significantly benefit from AHCL in terms of reducing the burden and risk of future complications (9).

Although the use of an AHCL in our cohort has led to a reduction in mean glucose and SD, the fact that the CV has not significantly changed may suggest that, relative to the mean glucose level, the spread or dispersion of glucose levels has not altered significantly.

This could potentially happen for several reasons. For instance, it is possible that while the mean glucose level and SD improved, they did so in a manner that maintained a relatively constant ratio, leading to a consistent CV. Another possibility is that the AHCL system has effectively reduced both extreme high and low glucose readings, causing improvements in mean glucose and SD, but still preserving some degree of glucose variability that is reflected in the CV. It is also worth noting that while we aim for lower variability in glucose management, some level of variability is natural and expected, especially in particular populations such as non-compliant adolescents, even with advanced management systems.

Safety is an essential component of AHCL technology in this population. No severe hypoglycemia was documented, which is consistent with other similar studies (4, 10); two episodes of moderate DKA occurred due to infusion set occlusion. Infusion set failure or occlusion is a well-documented complication of all insulin pump therapies, with higher rates seen in younger users (11). Therefore, frequent anticipatory education to avoid and manage infusion set issues remains crucial.

Limitations of our study include the small number of patients and the absence of a control group. The duration of the follow-up did not permit long-term conclusions; however, all enrolled adolescents will be followed for additional months to evaluate whether outcomes are confirmed.

For less complex T1D populations, closed-loop systems are already the gold standard therapeutic option (12). AHCL technology, combined with adequate training and clinical support, should now be considered a first-line approach for those with the most to gain, namely, non-compliant adolescents with T1D.

In conclusion, the pivotal role of AHCL technology in glucose control management is undeniable, demonstrating striking improvements even in non-compliant adolescents with T1D. Our study sheds new light on the immense potential of this technology, which could indeed be a game changer, a true turning point for those most in need of such assistance. Despite the challenging landscape of T1D management, particularly among non-compliant adolescents, our results point towards a path of improved quality of life and a future with fewer complications. This is not just a technological advancement, but a lifeline for these delicate subset of patients.

## Data availability statement

The original contributions presented in the study are included in the article/supplementary material. Further inquiries can be directed to the corresponding author.

## Ethics statement

The studies involving human participants were reviewed and approved by Comitato Etico - San Raffaele. The patients/participants provided their written informed consent to participate in this study.

## Author contributions

GF conceived the design of the work. VC collected data. GF, VC and AR drafted the article. All the authors contributed to the revision and approval of the final version.
